# Impact of ethnicity and socio-economic status on *Staphylococcus aureus* bacteremia incidence and mortality: a heavy burden in Indigenous Australians

**DOI:** 10.1186/1471-2334-12-249

**Published:** 2012-10-09

**Authors:** Steven Y C Tong, Sebastian J van Hal, Lloyd Einsiedel, Bart J Currie, John D Turnidge

**Affiliations:** 1 Tropical and Emerging Infectious Diseases Division, Menzies School of Health Research, Darwin, Northern Territory, Australia; 2 Department of Infectious Diseases, Royal Darwin Hospital, Darwin, Northern Territory, Australia; 3 Department of Microbiology & Infectious Diseases, Royal Prince Alfred Hospital, Sydney, NSW, Australia; 4 Antibiotic Resistance & Mobile Elements Group, Microbiology and Infectious Diseases Unit, School of Medicine, University of Western Sydney, Sydney, Australia; 5 Flinders University and the Northern Territory Clinical School, Adelaide, Australia; 6 SA Pathology, Women’s and Children’s Hospital, North Adelaide, and University of Adelaide, Adelaide, Australia

**Keywords:** *Staphylococcus aureus*, Bacteremia, Ethnicity, Socio-economic status, Indigenous

## Abstract

**Background:**

Investigations of the impact of ethnicity and socio-economic status on incidence and outcomes of *Staphylococcus aureus* bacteraemia are limited.

**Methods:**

We prospectively identified all *S. aureus* bacteraemia episodes in the Australian New Zealand Cooperative on Outcomes in Staphylococcal Sepsis cohort study between 2007 and 2010. We calculated population level incidence rates using regional postcodes and stratified the analysis by ethnicity, age and socio-economic status indexes.

**Results:**

There were 7539 episodes of *S. aureus* bacteraemia with an annual incidence of 11·2 episodes per 100,000 population. The age-adjusted incidence in the Indigenous population was 62·5 per 100,000 population with an age standardized incidence rate ratio of 5·9 compared to the non-Indigenous population and an incidence rate ratio of 29.2 for community-associated methicillin-resistant *S. aureus* (MRSA). Populations in the lowest socio-economic status quintile had an increased *S. aureus* bacteraemia incidence compared to higher quintiles. However, there was a disparity between Indigenous and non-Indigenous populations across all socio-economic status quintiles. The lower 30-day mortality for Indigenous patients (7%) compared to non-Indigenous patients (17%) was explained by differences in age.

**Conclusions:**

Indigenous Australians suffer from a higher rate of *S. aureus* bacteraemia than non-Indigenous Australians, particularly for community-associated MRSA. Ethnicity and socio-economic status had little impact on subsequent mortality, with other host factors contributing more significantly.

## Background

Ethnicity or race can influence the incidence and outcomes of infectious diseases. Compared to Caucasians, African-Americans and native Americans are 2·1 and 5·9 times more likely to acquire pneumococcal bacteremia respectively [1]. During the H1N1 2009 influenza A pandemic, Australian Indigenous populations were at increased risk of hospitalization [2]. In *Staphylococcus aureus* infections, African-Americans have an increased risk of acquiring community-associated methicillin-resistant *S. aureus* (MRSA) infections compared to Caucasians [3,4]. Similarly, bacteremia is more common in certain ethnic groups [5]. In Australia, Indigenous populations suffer from *S. aureus* bacteraemia (SAB) at rates significantly higher than non-Indigenous Australians in both northern and Central Australia [6-8].

Likewise, socio-economic status (SES) is known to impact infectious diseases rates through differences in living conditions, including household crowding and access to and use of washing and sanitation facilities, differences in comorbidities such as smoking, hazardous alcohol use and diabetes, access to health care facilities and differing health seeking behaviour. Previous studies have found lower income households [5] and those living in public housing complexes [4] to be at higher risk of acquiring community-associated MRSA infections or MRSA post-operative wound infections [9]; and compared to lower SES strata, SAB occurred significantly less frequently in higher SES strata [10]. Similarly, the prevalence of the dominant community-associated MRSA lineage in the United States (US), USA300 MRSA, is influenced by SES with the mean income of high prevalence neighbourhoods approximately US $15000 per annum less than low prevalence suburbs [11]. In Australia, a correlation between lower SES and the incidence of staphylococcal infections has been observed [7].

In contrast to the incidence of infections due to pathogens such as *S. aureus*, there is no consistent evidence that mortality is associated with SES. Although, a higher mortality due to sepsis has been detected in resource limited countries compared to wealthier countries, these data may be explained by differences in hospital access or care as opposed to SES [12]. In Central Australia, there were no differences between Indigenous and non-Indigenous patients in short term mortality; however, mortality for the subset of Indigenous patients presenting with more than one episode of blood stream infection was markedly increased [6]. Indigenous status was not found to be associated with survival using multivariate analysis in one study of SAB in Australia. However, this study included only a small number of Indigenous patients (n=53) [13]. In a recent study of sepsis from tropical northern Australia where *S. aureus* was the most common pathogen (25%), there was no significant difference in 28-day mortality rates between Indigenous and non-Indigenous patients, with multivariate analysis showing age to be a strong predictor of mortality [14].

Therefore, it remains unclear what contribution ethnicity per se makes to incidences and outcomes of SAB as ethnicity and SES are frequently interlinked. For example, in Australia and the United States, many Indigenous people live in remote communities or reservations where both poverty and remoteness impact on health and healthcare access. In addition, SES is a difficult variable to study as it is seldom collected or recorded in health studies. The aim of this study was to determine the impact of ethnicity and SES on the incidence and outcomes of SAB in a large nation-wide dataset of cases of SAB in Australia.

## Methods

All SAB episodes were prospectively identified from institutions participating in the Australian New Zealand Cooperative on Outcomes in Staphylococcal Sepsis [13] study between 2007 and 2010. Data were collected for each episode and included age, sex, postcode (zipcode equivalent), ethnicity, date of admission, date of discharge, principal diagnosis and principal antibiotic used for management and overall 30-day mortality. All data were entered through a web-based collection system with each anonymized entry uniquely identified at the participating institution to allow for follow-up and correction of discrepant results by regular audits.

Isolates were categorised into methicillin-susceptible *S. aureus* (MSSA) and MRSA episodes by their susceptibility pattern. MRSA was further characterized into probable community-associated MRSA (cMRSA) – resistant to ≤2 non-β-lactam antibiotics; and probable epidemic MRSA (eMRSA) comprising multi-resistant MRSA – resistant to ≥2 non-*B*-lactam antibiotics, and probable United Kingdom epidemic MRSA type 15 (E-MRSA 15) – resistant to ≤2 non-*B*-lactam antibiotics but resistant to ciprofloxacin ± erythromycin.

An episode of SAB was defined as a single positive blood culture bottle for *S. aureus* in a patient that had symptoms and signs consistent with an infection. A new episode in the same patient was recorded if the bacteraemia had cleared, but a further blood culture taken greater than 14 days after the initial positive culture was again positive. SAB episodes occurring ≥48 and <48 hours after hospital admission were considered nosocomial and community-onset bacteremias respectively. Community-onset episodes were deemed healthcare-associated if patients had ≥1 of the following healthcare-associated risk factors in the previous 12 months: hospitalisation, surgery, residency in a long term care facility or receipt of renal dialysis.

Denominator data were acquired from the Australian Bureau of Statistics (ABS) based on the 2006 census. Ethnicity, age and socio-economic indexes for area (SEIFA) were extracted by postcode [15]. The specific SEIFA index used was the “Index of Relative Socio-economic Advantage and Disadvantage” (IRSAD) which is a relative score constructed from data on income, educational level, employment status, occupation type, housing status, internet access, disability status, car ownership, and single parenthood status of a specific postcode compared to all other postcodes [15]. Indigenous status is not a variable used in the construction of the IRSAD. Deciles of the IRSAD, as provided and suggested for use by the ABS, were used for analysis. A lower IRSAD decile compared to a higher decile indicates a lower SES. Only SAB episodes occurring in postcodes represented in the ABS dataset were included in the analysis. Data collection was considered complete for the entire period for all institutions to calculate SAB incidences.

Statistical analysis was performed using Stata 11.2 (StatCorp, Texas, USA). Incidence rates and confidence intervals were calculated using the Poisson distribution. The Indigenous population was standardized against the non-Indigenous population age distribution using indirect methods and age standardized incidence rates with 95% confidence intervals were reported. For analysis of categorical and continuous data we used χ^2^, Fisher’s exact, analysis of variance, Student’s t-test and the Mann–Whitney U test as appropriate. A multivariate logistic regression model with 30-day mortality as the independent outcome was built with the initial inclusion of variables with a P<0·2 on univariate analysis and backwards stepwise elimination of variables using a likelihood ratio test to determine the statistical significance of candidate explanatory variables.

### Ethics

Approval to prospectively collect data was provided by the ethics committee of each participating institution: Australian Capital Territory – Canberra Hospital; New South Wales – Blacktown Hospital, Mount Druitt Hospital, Concord Hospital, John Hunter Hospital, Liverpool Hospital, Nepean Hospital, Royal North Shore Hospital, St Vincent’s Hospital, Westmead Hospital; Northern Territory – Royal Darwin Hospital, Alice Springs Hospital; Queensland – Ipswich Hospital, Princess Alexandra Hospital, Royal Brisbane and Women’s Hospital; South Australia – Royal Adelaide Hospital, Flinders Medical Centre, Women’s and Children’s Hospital; Tasmania – Royal Hobart Hospital; Victoria – The Alfred Hospital, Austin Health, Monash Medical Centre, Western Health; Western Australia – Fremantle Hospital, Royal Perth Hospital. Consent from each patient was not required as the collection of data was considered to be a clinical audit.

## Results

### Population level incidence of *S. aureus* bacteremia

There were 7633 SAB episodes identified from 24 participating institutions in Australia over the four years 2007–2010 from the Australian New Zealand Cooperative on Outcomes in Staphylococcal Sepsis study. 94 (1%) episodes were excluded as postcodes were incomplete or not represented in the 2006 Australian census. The proportion of Indigenous cases in the included dataset (536/7539 [7.1%]) was similar to that for the entire dataset (536/7633 [6.5%]). The final 7539 episodes for analysis occurred in an annualised population of 16,842,651 represented by 1258 postcodes from all States and Territories of Australia and comprising a non-Indigenous population of 16,453,420 and an Indigenous population of 389,230. The overall crude annual incidence was 11·2 (95% confidence [CI] 10.9, 11·5) SAB episodes per 100,000 population. There were 1256 deaths and an overall crude annual mortality rate of 1·9 (95% CI 1·8, 2·0) deaths per 100,000 population.

**Figure 1:**
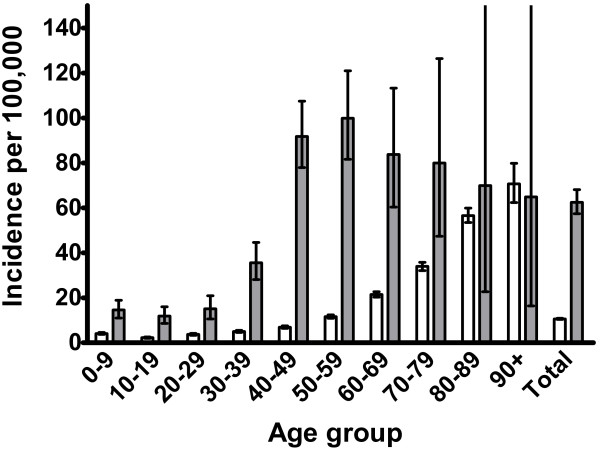
***S. aureus *bacteremia incidence rates by age and ethnicity.** Incidence rates are stratified by age comparing non-Indigenous (white columns) with Indigenous (grey columns) populations. The bars represent 95% confidence intervals calculated using a Poisson distribution. The “Total” column represents indirect age standardized incident rates.

There were 7003 non-Indigenous episodes and 536 Indigenous episodes. The crude non-Indigenous annual incidence was 10·6 and the age standardized Indigenous annual incidence was 62·5 per 100,000 population with an age standardized incidence rate ratio (IRR) of 5·9 (95% CI 5·4, 6·4) for Indigenous compared to non-Indigenous rates. Indigenous incidences were higher across all age strata except for those aged 80–89 and 90+ years (Figure 1). Notably, the peak incidence was in the 40–59 year old age groups for the Indigenous population, which was a markedly different pattern compared to the non-Indigenous population where incidence steadily increased with age. There were 1219 non-Indigenous deaths and 37 Indigenous deaths with an age standardized mortality ratio of 3·9 (95% CI 2·8, 5·4) for Indigenous compared to non-Indigenous rates.

**Figure 2:**
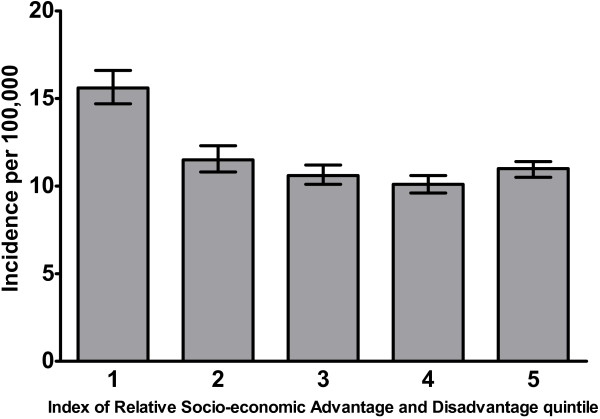
***S. aureus *bacteremia incidence rates by Index of Relative Socio-economic Advantage and Disadvantage quintiles.** The bars represent 95% confidence intervals calculated using a Poisson distribution.

**Figure 3:**
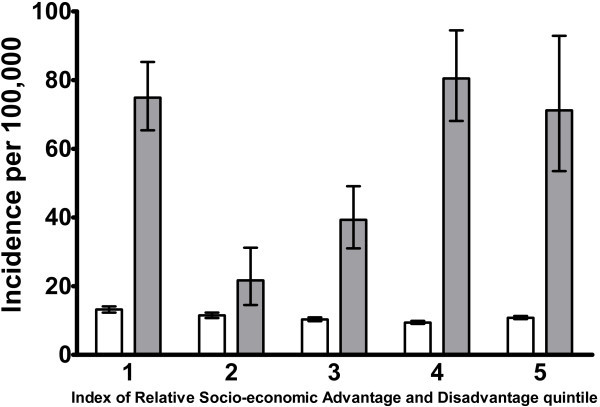
***S. aureus *bacteremia incidence rates by Index of Relative Socio-economic Advantage and Disadvantage quintiles and ethnicity.** Comparisons are between non-Indigenous (white columns) and Indigenous (grey columns) populations. Indirect age standardized incidence rates with 95% confidence intervals are reported.

We compared annual incidence rates stratified by SES as recorded by postcode using the “Index of Relative Socio-economic Advantage and Disadvantage” (IRSAD). This demonstrated that those in the lowest IRSAD quintile (i.e., lowest SES quintile) had a higher incidence than those in the upper four IRSAD quintiles (Figure 2) (P<0·001). To investigate if SES explained the differences in incidence rates of SAB between non-Indigenous and Indigenous populations we calculated age standardized incidence rates stratified by IRSAD quintiles (Figure 3). The overall pattern of higher incidence in the lowest IRSAD quintile was replicated when the non-Indigenous population alone was examined (Figure 3) (P<0·001). The numbers of cases within the quintiles for the Indigenous population were 227, 29, 77, 149 and 54 for quintiles one to five respectively. There was no clear trend in incidence across IRSAD quintiles for the Indigenous population. However, the differences between non-Indigenous and Indigenous populations existed across all IRSAD quintiles with age standardized IRRs ranging from 1·9 to 8.5 for Indigenous compared to non-Indigenous rates.

**Figure 4:**
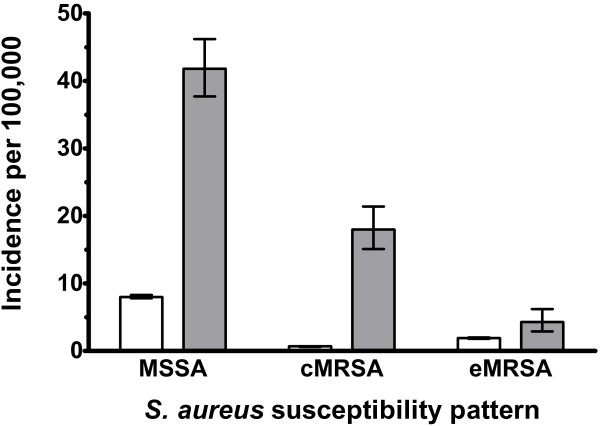
***S. aureus *bacteremia incidence rates stratified by susceptibility pattern and ethnicity.** Comparisons are between non-Indigenous (white columns) with Indigenous (grey columns) populations. Indirect age standardized incident rates with 95% confidence intervals are reported. MSSA, methicillin-susceptible *S. aureus*; cMRSA, community-associated methicillin-resistant *S. aureus*; eMRSA, epidemic MRSA.

We also compared age standardized IRRs for MSSA, cMRSA and eMRSA between non-Indigenous and Indigenous populations. Although the incidence rates were significantly higher for Indigenous populations for all three groups of *S. aureus* (Figure 4), this was most marked for cMRSA (IRR 29.2 [95% CI 24, 35]) compared to MSSA (IRR 5·2 [95% CI 4·7, 5·7]) and eMRSA (IRR 2·3 [95% CI 1·5, 3·3]) when comparing Indigenous to non-Indigenous rates.

### Ethnic differences for those with *S. aureus* bacteremia

**Figure 5:**
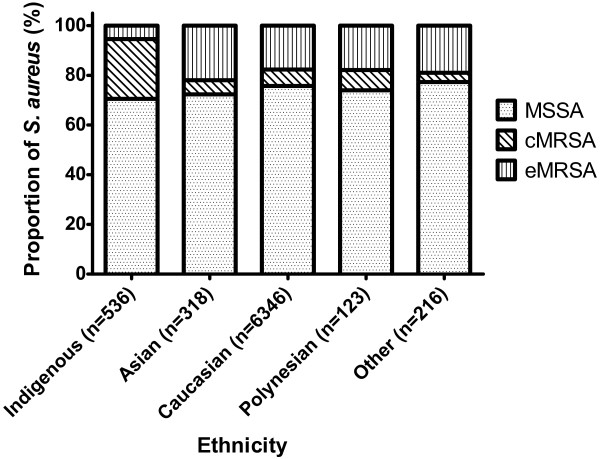
**The proportional distribution of susceptibility patterns of *S. aureus *bacteremia for different ethnicities.** MSSA, methicillin-susceptible *S. aureus*; cMRSA, community-associated methicillin-resistant *S. aureus*; eMRSA, epidemic MRSA.

We conducted a more detailed analysis of the 7539 episodes of SAB to investigate differences due to ethnicity. Of these episodes, the ethnic background was noted to be Indigenous for 536, Asian for 318, Caucasian for 6,346, Polynesian for 123, and ‘Other’ for 216. On univariate analysis, there were significant differences in age, IRSAD score, place of acquisition, receipt of dialysis, primary focus of infection, intensive care unit (ICU) admission, and mortality (Table 1). In particular, the 30-day mortality for Indigenous patients of 7% (37/536) was significantly less than that for non-Indigenous patients of 17% (1219/7003). In addition, a far greater proportion of SAB were due to cMRSA in the Indigenous (129/536, 24%) patients compared to other ethnicities (458/7003, 7%) (Figure 5). Indigenous patients also had the lowest mean IRSAD of all ethnic groups.

**Table 1:** **Comparison of characteristics of *S. aureus *bacteremia across ethnic populations**

	**Indigenous (n=536)**		**Asian (n=318)**		**Caucasian (n=6346)**		**Polynesian (n=123)**		**Other (n=216)**		***P***
**Demographic traits**											
Age (median)	44		55		65		48		58		<0.001^a^
Age (mean)	40	(20)	52	(25)	61	(23)	43	(22)	51	(25)	<0.001^b^
Sex (Male)	321	(60)	206	(65)	4179	(66)	80	(65)	144	(67)	0.09
IRSAD (mean)	4.6	(3.2)	6.9	(3.0)	6.7	(2.7)	4.7	(2.8)	6.3	(2.7)	<0.001^c^
**Acquistion**											<0.001^d^
CO	154	(29)	58	(19)	1235	(20)	23	(21)	33	(16)	
CO HCA	243	(46)	104	(34)	2517	(42)	48	(43)	86	(42)	
Nosocomial	131	(25)	141	(47)	2306	(38)	40	(36)	85	(42)	
**Risk factors**											
Dialysis	139	(26)	44	(14)	541	(9)	25	(20)	34	(16)	<0.001
IDU	30	(6)	17	(7)	369	(8)	5	(7)	11	(8)	0.86^e^
Device related	162	(32)	130	(43)	2145	(36)	39	(34)	80	(39)	0.02^f^
**Primary focus**											
Endocarditis	20	(4)	19	(6)	378	(6)	5	(4)	11	(5)	0.25
Bone/joint	91	(17)	40	(13)	782	(12)	21	(17)	22	(10)	0.01
SSTI	128	(24)	57	(18)	1152	(18)	27	(22)	51	(24)	0.005
**Outcomes**											
ICU	91	(17)	43	(14)	763	(12)	19	(15)	19	(9)	0.004
Mortality 7 days	15	(3)	24	(8)	636	(10)	8	(7)	9	(4)	<0.001
Mortality 30 days	37	(7)	49	(17)	1135	(19)	12	(12)	23	(12)	<0.001^g^

**Note.** Data are number (%) of patients and means (standard deviations). IRSAD, Index of Relative Socio-economic Advantage or Disadvantage; CO, community-onset; HCA, healthcare-associated; IDU, intravenous drug using; SSTI, skin and soft tissue infection; ICU, intensive care unit.

^**a**^ Kruskal-Wallis test; ^**b**^ Analysis of variance test (F=123.7, df=4); ^**c**^ Analysis of variance test (F=89.3, df=4); ^**d**^ χ^2^ test for 3x5 table. Missing data for Indigenous – 8, Asian – 15, Caucasian – 288, Polynesian – 12, Other – 12; ^**e**^ Missing data for Indigenous – 54, Asian – 75, Caucasian – 1474, Polynesian - 48, Other – 71; ^**f**^ Missing data for Indigenous – 23, Asian – 14, Caucasian – 370, Polynesian – 8, Other – 12; ^**g**^ Missing data for Indigenous – 17, Asian – 30, Caucasian – 415, Polynesian – 19, Other – 19.

### Predictors of mortality

Given the significant difference in 30-day mortality between Indigenous and non-Indigenous groups we looked for independent predictors of mortality on multivariate analysis. Independent predictors of mortality were age, healthcare-associated acquisition, the primary focus of infection, admission to ICU and the antibiotic treatment received (Table 2). Of note, neither ethnicity, SES using the IRSAD, nor the isolate susceptibility profile were independently associated with mortality. Thus, differences in mortality across the ethnicities are principally determined by the differences in age distributions of patients with SAB in the various ethnic groups as demonstrated by comparisons of mean and median ages (Table 1) and histograms of age (data not shown).

**Table 2:** **Multivariate analysis of variables associated with mortality in patients with *S. aureus *bacteremia**

**Variable**	**Unadjusted**			**Adjusted**		
	**OR (95% CI)**		**P**	**OR (95% CI)**		**P**
**Demographic characteristics**						
**Age**						
0-9	4.2	(1.7, 10.3)	0.002	5.1	(1.7, 15.3)	0.004
10-19	1.3	(0.4, 4.3)	0.682	2.4	(0.6, 10.0)	0.213
20-29 (reference)	1.0			1.0		
30-39	2.4	(1.0, 6.0)	0.059	2.7	(0.9, 8.4)	0.078
40-49	4.8	(2.1, 11.2)	<0.001	7.1	(2.5, 19.9)	<0.001
50-59	7.1	(3.1, 16.4)	<0.001	8.5	(3.1, 23.6)	<0.001
60-69	10.6	(4.7, 24.1)	<0.001	14.8	(5.4, 40.6)	<0.001
70-79	18.8	(8.3, 42.6)	<0.001	23.8	(8.7, 65.1)	<0.001
80-89	29.3	(12.9, 66.3)	<0.001	38.1	(13.9, 104)	<0.001
90+	46.2	(19.8, 108)	<0.001	54.4	(19.1, 154)	<0.001
**Sex**						
Female	1.0					
Male	1.0	(0.9, 1.2)	0.838			
**Ethnicity**						
Indigenous	1.0					
Asian	2.7	(1.7, 4.2)	<0.001			
Caucasian	3.1	(2.2, 4.3)	<0.001			
Polynesian	1.7	(0.9, 3.4)	0.131			
Other	1.7	(1.0, 3.0)	0.052			
**IRSAD quintile**						
1	1.0					
2	1.3	(1.0, 1.7)	0.033			
3	1.3	(1.0, 1.6)	0.050			
4	1.2	(1.0, 1.5)	0.091			
5	1.3	(1.0, 1.6)	0.026			
**Risk factors and Acquisition**						
**Dialysis**						
No	1.0					
Yes	0.7	(0.5, 0.8)	<0.001			
**IDU**						
No	1.0					
Yes	0.3	(0.2, 0.4)	<0.001			
**Acquisition**						
Community onset	1.0					
Community onset HCA	2.1	(1.7, 2.5)	<0.001	1.4	(1.1,1.7)	0.011
Nosocomial	2.4	(2.0, 2.9)	<0.001	1.5	(1.2,1.9)	0.001
**Microbiology and antibiotics**						
**Susceptibility**						
MSSA	1.0					
cMRSA	1.3	(1.0, 1.6)	0.025			
eMRSA	2.0	(1.7, 2.3)	<0.001			
**Antibiotic treatment**						
β-lactam	1.0					
Glycopeptide	2.0	(1.7, 2.3)	<0.001	1.5	(1.3, 1.8)	<0.001
Other	2.1	(1.6, 2.8)	<0.001	1.6	(1.2, 2.2)	0.002
**Clinical infection**						
**Primary focus**						
SSTI	1.0			1.0		
Device with 2° infection	1.8	(1.2, 2.7)	0.003	1.9	(1.3, 2.9)	0.003
Device with no 2° infection	0.8	(0.7, 1.0)	0.118	0.9	(0.7, 1.2)	0.600
IE Left sided	1.5	(1.0, 2.1)	0.025	2.2	(1.5, 3.2)	<0.001
IE Right sided	0.6	(0.3, 1.1)	0.092	1.4	(0.7, 3.0)	0.308
No focus	1.9	(1.6, 2.4)	<0.001	1.4	(1.1, 1.9)	0.011
Bone/joint	0.6	(0.4, 0.8)	<0.001	0.8	(0.6, 1.1)	0.228
Respiratory	3.7	(2.9, 4.8)	<0.001	3.6	(2.7, 4.8)	<0.001
Sepsis	4.4	(3.5, 5.5)	<0.001	4.0	(3.1, 5.3)	<0.001
**ICU**						
No	1.0			1.0		
Yes	1.8	(1.5, 2.1)	<0.001	2.2	(1.8, 2.7)	<0.001

**Note.** Adjusted odds ratios and P values are only displayed if that variable was significant in the final multivariate model. OR, odds ratio; CI, confidence interval; IRSAD, Index of Relative Socio-economic Advantage or Disadvantage; HCA, healthcare-associated; MSSA, methicillin-susceptible *S. aureus*; cMRSA, community-associated methicillin-resistant *S. aureus*; eMRSA, epidemic MRSA; SSTI, skin and soft tissue infection; IE, infective endocarditis; ICU, intensive care unit.

## Discussion

In analysing an Australia wide dataset of 7539 episodes of SAB we found a disturbingly large disparity in the population incidence of SAB between the non-Indigenous and Indigenous populations of Australia with an age-adjusted incidence rate ratio of 5·9 for Indigenous compared to non-Indigenous populations. Increased incidence of SAB according to ethnicity has previously been noted [7,8,16-18], however, this study is unique in the size of the disparity observed and the finding that the disparity is particularly marked for community-associated MRSA. There are key public health implications arising from these findings regarding strategies to prevent staphylococcal infections in Indigenous communities.

Although focused on MRSA, previous studies have shown high rates of staphylococcal skin and soft tissue infections [19] in many Indigenous communities around the world [20]. In Australian Indigenous populations, the high burden of staphylococcal skin disease appears to translate directly to higher rates of SAB. Risk factors are highly prevalent for community-associated *S. aureus* infections in Indigenous communities [19] and include those typically quoted for community-associated MRSA such as close skin-to-skin contact, openings in the skin such as cuts or abrasions, contaminated items and surfaces, crowded living conditions, and poor hygiene (the five Cs) ( http://www.cdc.gov/mrsa/riskfactors/index.html). Australian Indigenous remote households are often crowded (mean of 3·2 people per bedroom) [21] with inadequate facilities for personal hygiene [22]. There are high rates of skin infections with approximately 70% of children in remote communities presenting to the local health clinic with scabies infestation and impetigo by the age of one year [23]. Such findings are mirrored in other Indigenous populations [24].

Concomitant with the prevalence of these risk factors in Indigenous communities, and therefore the likely widespread use of antibiotics, we noted a considerably larger incidence rate ratio for Indigenous compared to non-Indigenous populations for cMRSA bacteremia (IRR 29.2), as contrasted with MSSA (IRR 5·2) and eMRSA (IRR 2·3). This finding is supportive of a previous hypothesis that community-associated MRSA may be emerging from within, and subsequently spreading outside of, Indigenous populations [19]. Previous studies have also noted an increased risk for MRSA infections [25] and bacteraemia [26] in Indigenous patients. Prevention of staphylococcal infections in general and community-associated MRSA specifically will thus require a multifaceted approach that includes education about the five Cs as knowledge and awareness of hygiene remains poor [27]. In addition, addressing issues of housing and disadvantage is critical in limiting the spread of community-associated MRSA [21,27]. In the meantime, there is emerging evidence to suggest that regional community health provider based initiatives in Indigenous communities can be successful in reducing scabies and skin infections [28] and that the use of trimethoprim-sulfamethoxazole rather than benzathine penicillin to treat impetigo, which is typically caused by both streptococci and staphylococci in these settings, is a promising approach in regions with high rates of community-associated MRSA [29].

For the overall dataset and also the large subset of the non-Indigenous population from across Australia with 7003 episodes of SAB, there was a higher SAB incidence in the lowest IRSAD quintile compared to the higher IRSAD quintiles. This is in agreement with a recent analysis of *S. aureus* from the tropical north of Australia where there was a strong correlation between incident isolation of *S. aureus* and measures of both regional socioeconomic disadvantage and remoteness [7]. There was no clear pattern in the Indigenous population for SAB incidence across IRSAD quintiles, but when we compared non-Indigenous and Indigenous populations stratified by IRSAD quintiles, significant disparities continued to be present between the ethnic groups across all IRSAD quintiles. It remains unclear what determines this ethnic disparity and particularly whether unmeasured aspects of ethnicity and socioeconomic status may contribute to this increased risk. The lack of a pattern across IRSAD quintiles within the Indigenous population may also suggest problems with the IRSAD measure or inaccuracies due to the relatively small sample size within some of the quintiles. Nevertheless, it is likely that the IRSAD score, even at the finest of level of postcode (with a 2006 estimated average population of 7993 ± 10510), fails to reflect aspects of the social disadvantage of many Indigenous communities, particularly in the sparsely populated regions of northern and Central Australia where the majority of the Indigenous cases came from. Indeed, the component factors for the IRSAD and other measures of SES sought in census surveys do not reflect the risk factors for staphylococcal skin infections. In particular, the impact of household crowding, the lack of running water and personal hygiene facilities and the impact of remote disadvantage are not well captured.

Although the incidence of SAB in the Indigenous population was higher, once diagnosed with SAB, Indigenous patients had a surprisingly lower mortality rate than non-Indigenous populations on univariate analysis. This is most likely explained by the fact that Indigenous patients acquired their SAB at a younger age than non-Indigenous patients. This is supported by a recent study of sepsis in tropical northern Australia, where incidences of sepsis and severe sepsis were much higher for Indigenous patients, but there was no difference in mortality rates, with age and living in residential care being the major predictors for mortality [14]. Notably, SES was not significantly associated with mortality for either Indigenous or non-Indigenous patients.

There are several limitations to this study. First, for calculation of incidence rates we assumed that all episodes from postcodes included in the study have been captured. This is unlikely to be the case, particularly as not all hospitals servicing these postcodes participated in the study. Thus the reported incidence rates are likely to underestimate the true incidence. However, this should not significantly impact upon the key finding of a relative increased incidence in the Indigenous population. Second, no data on comorbidities was collected and this is likely to impact upon mortality. An indication of this is that the presence of healthcare-associated risk factors was found to be independently associated with mortality and may be acting as a proxy for comorbidities. There was also missing data for some variables (such as for injection drug use) as presented in Table 1. Third, we were not able to assess the potential role of host genetic factors [30] in relation to both incidence and mortality. Fourth, as noted already use of postcodes for SES analysis may well have masked real SES differences between Indigenous and non-Indigenous patients, especially for some locations with particularly high rates of SAB in Indigenous patients [66]. It is possible that inclusion of such details would further explain the disparity in incidence rates between the Indigenous and non-Indigenous populations. Additionally, there is likely to be significant heterogeneity in SES within postcodes and future studies should consider accounting for such heterogeneity by collecting SES data on an individual case level. Finally, the categorization of MRSA by antibiogram into probable cMRSA and probable eMRSA is not as accurate as using genotyping techniques such as *spa*, multilocus sequence or SCC*mec* typing and may have resulted in some cases of misclassification.

## Conclusions

Our prospective study identified more than 7500 SAB episodes occurring in population of 16,453,420 resulting in a crude annual incidence of 11·2 episodes per 100,000 population. Overall Indigenous Australians were 5·9 times more likely to have a SAB, especially community-associated MRSA episodes (29.2 times) compared to non-Indigenous Australians. Populations in the lowest SES quintile had an increased SAB incidence compared to higher quintiles. However, there remained a disparity between Indigenous and non-Indigenous populations across all SES quintiles. Thus our study provides robust evidence that Indigenous populations and lower SES populations are at increased risk of SAB. Furthermore, standard measures of SES do not explain the disparity in rates between Indigenous and non-Indigenous populations.

## Competing interests

The authors declare that they have no competing interests.

## Authors’ contributions

ST, SVH study design, analysis and manuscript preparation. LE, BC and JT manuscript review and finalisation. All authors read and approved the final manuscript.

## Funding statement

This work was supported in part by an Australian National Health and Medical Research Council Postdoctoral Training Fellowship (508829) to ST.

## Pre-publication history

The pre-publication history for this paper can be accessed here:

http://www.biomedcentral.com/1471-2334/12/249/prepub
